# Interventions for Caregivers Caring for a Family Member With Advanced Illness at Home: A Systematic Review

**DOI:** 10.1111/nhs.70196

**Published:** 2025-08-17

**Authors:** Leire Sevillano‐Garayoa, Maddi Olano‐Lizarraga, Jesús Martín‐Martín

**Affiliations:** ^1^ Nursing Care for Adult Patients Department, Faculty of Nursing University of Navarra Pamplona Spain; ^2^ Innovation for a Person‐Centred Care Research Group, Faculty of Nursing University of Navarra Pamplona Spain; ^3^ Alpha Gamma Alpha Chapter #623 Navarra Spain; ^4^ Navarra's Health Research Institute (IdiSNA) Pamplona Spain

**Keywords:** advanced illness, family, home care, interventions, systematic review

## Abstract

The rising prevalence of advanced illness poses growing challenges for family caregivers, requiring healthcare professionals to address an increasing array of caregiver needs. This systematic review explores the impact of at‐home interventions for caregivers supporting individuals with advanced illness, examining intervention characteristics and methodological quality. Quantitative studies published over the past 5 years (2019–2024) were sourced from PubMed, PsycINFO, CINAHL, Scopus, and Cochrane Library. Intervention data, including modality, components, timing, providers, recipients, and caregiver outcomes, were systematically reviewed. Methodological quality was assessed using Cochrane RoB 2 and ROBINS‐I. From 16 studies, key caregiver needs such as depression, anxiety, distress, quality of life, burden, and caregiving self‐efficacy emerged as primary areas addressed. The majority of interventions were face‐to‐face, nurse‐led, and aimed at primary caregivers. Findings underscore the importance of personalized interventions that consider caregivers' unique responses and actively involve them in design. Nurses play a crucial role in leading these interventions, positioning them as central to enhancing caregiver support in home settings.

**Summary**

- What does this paper add?
  ○ This review identifies and compares home‐based interventions for family caregivers of people with advanced illness, highlighting the different formats, durations, professional roles involved, and outcomes targeted across 16 studies.
  ○ It shows that although many interventions aim to improve caregiver outcomes such as depression, anxiety, distress, quality of life, caregiver burden, and caregiving self‐efficacy or competence, their overall effectiveness is modest and often not sustained over time.
  ○ It underscores the importance of designing flexible, personalized interventions that are developed in partnership with caregivers themselves, rather than applying pre‐designed solutions that may not meet their specific needs.

## 1 Background

Across Europe, more than 4.4 million people die each year from conditions associated with serious health‐related suffering, often in the advanced stages of chronic illness. This represents a significant challenge for health systems in meeting the needs of individuals with advanced illness and their families (Garralda et al. 2025). Advanced illness refers to a spectrum of clinical conditions marked by progressive decline in health and functional status, limited response to curative treatments, and increasing care complexity (Hopp and Nedjat‐Haiem 2019; Sercu et al. 2018). It encompasses the later stages of chronic illness—including terminal and end‐of‐life phases—and reflects a trajectory in which symptom burden intensifies and quality of life becomes the central focus of care (Brar et al. 2023; Kuharic et al. 2024). Rather than being restricted to a specific moment near death, advanced illness captures the evolving and overlapping nature of serious illness, promoting a shared understanding that supports continuity of care across settings and over time (Leung and Zehm 2022; Garcia et al. 2023). While these types of deaths predominantly occur in healthcare facilities, there is an increasing trend of dying in the comfort of one's own home (Morris et al. 2015). In fact, many deaths that occur in hospitals go against the wishes of these patients, as most of them would prefer to die at home surrounded by their family and loved ones (Ali et al. 2019; Higginson et al. 2017). Hence, there has been a steady increase in the availability of end‐of‐life care at home in recent years (Arias‐Casais et al. 2020; Kjellstadli et al. 2018).

In this context, it has been shown that the likelihood that patients with advanced illness will spend the end of their lives at home mainly depends on the effort and capacity of the family members in charge of their care (Dowd et al. 2023; Martín‐Martín et al. 2022). In this regard, family members are responsible for providing most of the physical and emotional care, symptom management, and organization and coordination of health services on behalf of the patient (Zavagli et al. 2019). Thus, the family is the backbone of end‐of‐life care (Stajduhar 2013), providing approximately 80% of at‐home care for advanced patients (Martín‐Martín et al. 2016; Morris et al. 2015).

Although home care has benefits for patients and families by fostering feelings of freedom (Collier et al. 2015) and providing a deeper sense of human existence and appreciation of life (Akpan‐Idiok and Anarado 2014), it has also been shown to have a significant impact on the physical, psychological, and emotional well‐being of families (Martín‐Martín et al. 2022). The entire family faces challenges and crises when dealing with numerous issues in managing the life of their loved one, including symptom management and hospital admissions (Martín‐Martín et al. 2016). In addition, evidence has shown that family members feel unprepared to take on these responsibilities and experience a significant lack of confidence, as they are unaware of the caregiving tasks and challenges of assuming the role of caregiver (Mason and Hodgkin 2019; Soroka et al. 2018).

These circumstances underscore the importance of health professionals caring for families at home. Supporting and responding to the needs of family members are crucial to enabling them to sustainably provide home care and satisfy their preferences for care in this setting (Ewing et al. 2018; Hardy 2018). However, family‐centred practices, on many occasions, remain a clinical ideal since many health professionals continue to focus their attention exclusively on the patient, forgetting that families also want and need to be cared for (Ellington et al. 2018). On the other hand, professionals sometimes do not know how to involve the family in caring for their loved ones (Lee et al. 2014); this means that family care remains a current challenge in end‐of‐life care at home.

Previous studies have analyzed the provision of support for family caregivers without delimiting the place where the care was provided (Chi et al. 2016; Harding et al. 2012) and have focused on a single professional group (e.g., nurses) (Becqué et al. 2019) or a specific advanced illness (e.g., terminal cancer) (Ahn et al. 2020), limiting the generalizability of the findings. In response to the increasing demands and needs expressed by family caregivers who assist a relative with advanced illness at home, this systematic review was undertaken with the primary objective of identifying and evaluating the impact of existing interventions within this context, regardless of the type of advanced illness of the loved one or the disciplines involved in their implementation. Additionally, the secondary objectives were to analyze the methodological quality of the existing interventions and to examine the specific characteristics and components of these interventions.

## 2 Methods

The study was preregistered with the PROSPERO international prospective register of systematic reviews (under registration number CRD42024594627) and followed the Preferred Reporting Items for Systematic Reviews and Meta‐Analyses (PRISMA) protocol (Page et al. 2021).

### 2.1 Search Strategy

**FIGURE 1:**
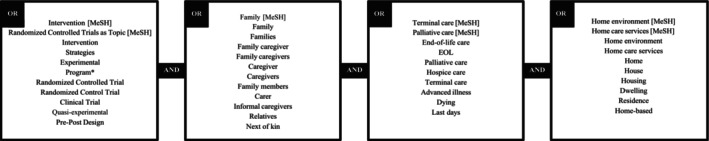
Search strategy. Representation of the search strategy conducted. The key terms used were *“interventions”*, *“family caregiver”*, *“advanced illness”*, and *“home”*, along with their synonyms, appropriately combined using boolean operators *“AND”* and *“OR”*. This search strategy was replicated across the different databases consulted (PubMed, PsycINFO, CINAHL, Scopus, and Cochrane Library).

The Population, Interventions, Comparison, and Outcomes (PICO) method was used to formulate the research question and guide the identification of search terms (Eriksen and Frandsen 2018). As specified in the registered protocol, the outcome domains were defined a priori within this framework, informed by the research team's previous qualitative studies on the phenomenon under investigation (Martín‐Martín et al. 2016; Martín‐Martín et al. 2022; Martín‐Martín et al. 2025). However, this a priori specification did not preclude the identification of additional outcome domains that emerged inductively from the included studies, thereby allowing the review to capture a broader range of effects reported in the literature. Guided by the PICO structure, the literature search was conducted between March and April 2023, and the data were updated in August 2024. Five electronic databases (PubMed, CINAHL, PsycINFO, Cochrane Library, and Scopus) were searched with the keywords *“interventions*,*” “family caregiver*,*” “advanced illness*,*”* and *“home”* identified from MeSH terms, a thesaurus, and the literature. These words and their synonyms were combined with the Booleans *“AND”* and *“OR*,*”* as shown in Figure 1 (see Appendix A for the search string).

### 2.2 Eligibility Criteria and Study Selection

**FIGURE 2:**
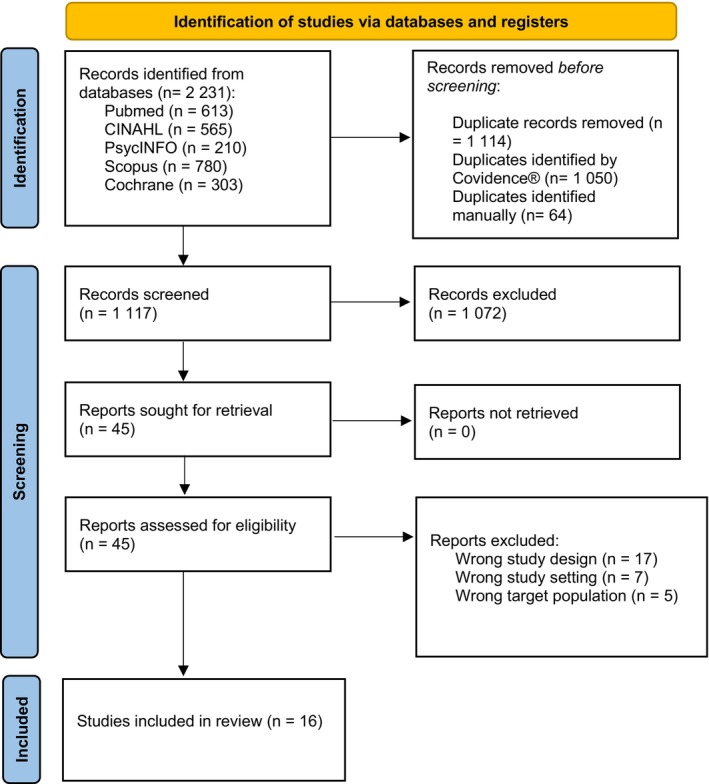
Flow diagram. Detailed representation of the entire selection process for the included articles.

The Mendeley reference management tool was used for data management throughout the systematic review. All citations were imported, and duplicates were removed using Covidence. Two researchers independently screened records by publication date and language, assessed titles and abstracts, and evaluated full texts for eligibility. Articles were selected according to predefined inclusion and exclusion criteria (Table 1). Any discrepancies were discussed in group meetings involving all three authors until consensus was reached. The PRISMA flow diagram was applied to document and report the selection process (see Figure 2).

**TABLE 1:** Eligibility criteria.

Inclusion criteria	Exclusion criteria
Studies about interventions for family caregivers of adult patients with advanced disease (an active and progressive disease with an unfavorable prognosis and no known cure) at home.	Studies on interventions for family caregivers of pediatric patients with advanced and terminal illness.
Interventions are carried out by different disciplines (e.g., medicine, nursing, psychology, etc.).	Studies on interventions for family caregivers aimed at achieving health outcomes in patients.
Quantitative studies.	Studies on interventions whose population are bereaved family caregivers.
Date: January 2019–August 2024	Gray reading and doctoral theses.
Language: Spanish, Portuguese, Italian, and English.	—

### 2.3 Data Extraction

After screening, identifying, and assessing the quality of the selected studies, data were extracted using a pre‐prepared form by researchers. The extracted information included authors and date of the study, country and place where the study was conducted, characteristics of the participants, study design, conceptual framework (understood as the theoretical model or rationale explicitly used to guide the design of the intervention and to explain its expected mechanisms of action (Van der Waldt 2024)), type of intervention (duration, mode of delivery, and the providers and recipients of the interventions), outcomes (variables measured, instruments used, efficacy of the interventions and conclusions of the original authors), and quality of the selected studies (see Table 2). The data extraction process was performed independently by the first (LSG) and third authors (JMM). A final check of the data was made by the second author (MOL).

**TABLE 2:** Summary of results.

Author (Year) Country	Aim(s)	Study design	Intervention	Control	Outcomes measurement tools	Main results	Risk of bias
Boyko et al. (2021) Canada	To determine whether the online Home Caregiver Support Program (HCSP) is effective in improving caregivers' perceived competence in addressing the challenges faced by those caring for the chronically or terminally ill.	Quasi‐experimental study	The HCSP consists of seven modules: introduction, physical needs (×2), emotional and psychological needs (×2), social and information needs, and spiritual needs.	—	Pre‐module competency survey: rate your perceived competency, according to the module, using 5 questions scored on a 5‐point Likert scale. Post‐module skills survey: the same survey as the one answered before the module.	Introduction (Q1: *W* = 68, *p* < 0.0001; Q2: *W* = 52.5, *p* = 0.0001; Q3: *W* = 76.5, *p* < 0.0001; Q4: *W* = 34.5, *p* = 0.0044; Q5: *W* = 50, *p* = 0.0028) Physical needs: Module 1 (Q1: W = 68, *p* < 0.0001; Q2: *W* = 63, *p* = 0.0003; Q3: *W* = 85.5, *p* < 0.0001; Q4: *W* = 45.5, *p* = 0.0002; Q5: *W* = 52.5, *p* = 0.0001) Module 2 (Q1: W = 68, *p* < 0.0001; Q2: *W* = 60, *p* < 0.0001; Q3: *W* = 68, *p* < 0.0001; Q4: *W* = 60, *p* < 0.0001; Q5: *W* = 52.5, *p* = 0.0001) Emotional and Psychological needs: Module 1 (Q1: W = 76.5, *p* < 0.0001; Q2: *W* = 76.5, *p* < 0.0001; Q3: *W* = 68, *p* < 0.0001; Q4: *W* = 33, *p* = 0.001; Q5: *W* = 60, *p* < 0.0001) Module 2 (Q1: W = 85.5, *p* < 0.0001; Q2: *W* = 68, *p* < 0.0001; Q3: *W* = 39, *p* = 0.0005; Q4: *W* = 76.5, *p* < 0.0001; Q5: *W* = 60, *p* < 0.0001) Social and information needs (Q1: W = 76.5, *p* < 0.0001; Q2: *W* = 60, *p* < 0.0001; Q3: *W* = 85.5, *p* < 0.0001; Q4: *W* = 77.5, *p* = 0.0007; Q5: *W* = 95, *p* < 0.0001) Spitirual needs (Q1: W = 63.5, *p* = 0.0003; Q2: *W* = 79.5, *p* < 0.0001; Q3: *W* = 48, *p* = 0.0011; Q4: *W* = 52.5, *p* = 0.0001; Q5: *W* = 49.5, *p* = 0.0007)	Moderate (ROBINS‐I)
Chow et al. (2024) China	To evaluate the differential effects of an integrated community‐based end‐of‐life support team (ICEST) model for terminally ill older adults on spousal and adult‐child caregivers.	Pre‐post study	Integrated end‐of‐life support team (ICEST) providing psychoeducation, counseling, caregiving skills training, and family‐centered activities.	—	Chinese version of the modified Caregiver Strain Index (C‐MCSI)	– Caregiving strain −2.7 (SD = 5.1) *t* = −8.2; *d* = −0.5 – Depression: −0.5 (SD = 1.3); *t* = −5.8; *d* = −0.4 – Perceived support: +0.5 (SD = 0.9); *t* = 9.1; *d* = 0.6 – Information provision: +0.6 (SD = 0.9); *t* = 10.8; *d* = 0.7 – Agreement on care plans: +0.3 (SD = 1.2); *t* = 3.6; *d* = 0.2 – Intimacy: +0.3 (SD = 0.8); *t* = 6.5; *d* = 0.4	Moderate (ROBINS‐I)
Dionne‐Odom et al. (2022) USA	Evaluate the feasibility, acceptability, and potential efficacy of ENABLE Cornerstone.	Pilot RCT	Six weekly individual telephone counseling sessions (20–60 min): (1) establishing rapport; (2) stress strategies; (3) asking for help in completing tasks; (4) health information; (5) self‐care dimension; (6) decision making. Subsequently, monthly follow‐up calls for 24 weeks to address any new or ongoing issues.	Usual care	– 14‐item Hospital Anxiety and Depression Scale (HADS). – 35‐item Caregiver Quality of Life‐Cancer assessment.	– Anxiety: At 8 weeks: −0.21 (95% CI: −0.69 to 0.27); *d* = −0.21 At 24 weeks: −1.94 (95% CI: −0.98 to 0.11); *d* = −0.44 – Distress: At 8 weeks: −0.08 (95% CI: −0.57 to 0.41); *d* = −0.08 At 24 weeks: −2.29 (95% CI: −0.88 to 0.24); *d* = −0.32 – Quality of life: At 8 weeks: −2.07 (95% CI: −5.45 to 1.32); *d* = −0.24 At 24 weeks: −1.56 (95% CI: −6.23 to 3.12); *d* = −0.07	Some concerns (RoB 2)
Fleisher et al. (2023) USA	Respond to the needs of patients with advanced Parkinson's disease and their families through a program that combines home visits and caregiver mentoring.	Non‐Randomized Clinical Trial (RCT)	IN‐HOME‐PD: dyads receive home visits by a nurse and social worker accompanied by a telehealth connection with a Parkinson's specialist. Share the Care: 16‐week nested caregiver mentoring intervention.	Parkinson's patients and their caregivers receive brief annual assessments while receiving regular care at a Parkinson's Foundation Center of Excellence.	– Multidimensional Caregiver Strain Index (MCSI). – HADS. – Caregiver Self‐Efficacy Scale (CSES).	– Total caregiver strain: baseline 23.34 (SD 9.43) → 1 year 24.32 (SD 9.72); *p* = 0.51 – Anxiety: Visit 1: 7.07 (SD 4.1) → Visit 4: 5.96 (SD 3.95); *p* = 0.049 – Depression: Visit 1: 4.95 (SD 3.46) → Visit 4: 4.96 (SD 3.54); *p* = 0.24 – Self‐efficacy—Community support services use: Visit 1: 25.54 (SD 9.43) → Visit 4: 26.1 (SD 9.05); *p* = 0.62	Serious (ROBINS‐I)
Gregory and Gellis (2020) USA	To examine the effectiveness of Brief Problem‐solving Therapy for Hospice (PST‐Hospice) as a systematic approach to reduce caregivers' distress and improve their coping ability.	Pilot RCT	PST‐Hospice sessions per week plus usual care.	An educational brochure on how to cope with care was sent via email + usual care (interdisciplinary palliative care team offering support and care to the patient‐family according to an individualized care plan).	– Caregiver Quality of Life Index–Cancer (CQOLC). – Social Problem‐Solving Inventory‐Revised Short Form (SPSI‐R Short Form).	– Depression: *F* 5.79, *p* < 0.01 – Quality of Life: *F* 6.92, *p* < 0.001 – Problem‐solving abilities: Positive Problem Orientation (*F* 3.14, *p* < 0.06) and rational problem solving (*F* 3.83, *p* < 0.08)	Some concerns (RoB 2)
Ito and Tadaka (2022) Japan	To determine the effect of the ONDIARY program on the quality of life of caregivers of advanced cancer patients in home‐based palliative care settings.	Quasi‐experimental study	ONDIARY (daily guided reflection and writing in an online diary) plus usual care.	Usual care (assistance with activities of daily living and medical care by a multidisciplinary team).	– Japanese version of the Caregiver Quality of Life Index‐Cancer (CQOLC). – Six‐item Kessler Psychological Distress Scale (K6).	‐ Quality of life: Intervention: baseline 69.1 (SD 17.9) → follow‐up 70.1 (SD 19.6) Control: baseline 73.6 (SD 14.1) → follow‐up 67.6 (SD 15.0) Group × Time interaction: *p* = 0.003 ‐ Depression: Intervention: baseline 9.0 (SD 6.8) → follow‐up 10.0 (SD 6.7) Control: baseline 9.0 (SD 6.0) → follow‐up 9.9 (SD 6.3) Group × Time interaction: *p* = 0.933	Moderate (ROBINS‐I)
Lund et al. (2020) Denmark	To determine the impact of the CSNAT‐I on caregivers of patients enrolled in specialized palliative care at home in Denmark.	Randomized cluster study	CSNAT‐I consists of two parts: the tool covers caregivers' needs for care and self‐care and is integrated into a framework of person‐centred care directed by the caregiver.	—	– Family Appraisal of Caregiving Questionnaire for Palliative Care (FACQ‐PC) – Cancer Caregiving Tasks, Consequences and Needs Questionnaire (CaTCoN). – EORTC Quality of Life Questionnaire Core 30 (EORTC QLQ‐ C30).	T1 (2 weeks later): – Caregiver strain (*d* = 0.15; *p* = 0.1875) – Distress (*d* = 0.12; *p* = 0.0178) – Positive caregiving appraisals (*d* = 0.08; *p* = 0.6792) – Quality of information and communication (*d* = 0.57; *p* < 0.0001) – Quality of life (*d* = 0.03; *p* = 0.9274) T2 (4 weeks later): – Caregiver strain (*d* = 0.19; *p* = 0.1533) – Distress (*p* = 0.4424; *d* = 0.04) – Positive caregiving appraisals (*d* = 0.12; *p* = 0.4409) – Quality of information and communication (*d* = 0.88; *p* < 0.0001) – Quality of life (*d* = 0.11; *p* = 0.7534)	Some concerns (RoB 2)
Mooney et al. (2023) USA	To determine the effectiveness of the Symptom Care at Home (SCH) intervention in reducing caregiver burden.	Multicentre RCT	SCH is a digital tool that assesses daily self‐reported symptoms (anxiety, depression, fatigue, sleep disorders and caregiving interference with usual life) and, based on algorithms, provides automated coaching messages for self‐help.	Usual palliative care and collection of caregiver symptoms but not addressing them.	Caregiver Burden measure: A proprietary scale from 0 to 10 is used to measure the five items: anxiety, depression, fatigue, sleep disturbances, and interference of caregiving with usual life.	– Total Caregiver Burden: *p* < 0.001; 95% CI: (5.67, 12.97); *d* = 0.44 – Mood Component: *p* < 0.001; 95% CI: (3.31, 9.40); *d* = 0.33 – Vitality Component: *p* < 0.001; 95% CI: (6.37, 13.81); *d =* 0.47	Some concerns (RoB 2)
Norinder et al. (2024) Sweden	To explore the effects of the Carer Support Needs Assessment Tool Intervention (CSNAT‐I) on preparedness, caregiver burden, and quality of life among family caregivers in specialized home care.	Pre‐post study	CSNAT‐I consist of two parts: a validated assessment tool (CSNAT) that is structures around support domains and a 5‐stage person‐centered process: (1) introduce CSNAT‐I; (2) family caregiver considers needs; (3) assessment conversation; (4) shared action plan; (5) shared review.	—	– Preparedness for Caregiving Scale (PCS) – Caregiver Burden Scale (CBS) – Quality of Life in Life‐Threatening Illness—Family Version (QOLLTI‐F)	– Preparedness for caregiving: Mdn 18 (15–20) to 20 (17–22), *p* =. 002. – Caregiver burden: environment domain [Mdn 1.7 (1.5–2.3) to 1.7 (1.3–2.0), *p* = 0.029]; general strain domain [Mdn 2.4 (1.8–2.6) to 2.3 (1.8–2.6), *p* = 0.740]; isolation domain [Mdn 2.7 (2.0–3.0) to 2.7 (2.0–3.0), *p* = 0.362]; disappointment domain [Mdn 2.2 (2.0–2.6) to 2.2 (2.0–2.6), *p* = 0.6339]; emotional involvement domain [Mdn 1.7 (1.0–2.3) to 1.7 (1.0–2.0), *p* = 0.247] – Quality of life: overall [Mdn 6.0 (5.0–8.0) to 6.0 (5.0–8.0), *p* = 0.645]; all domains *p* > 0.4	Moderate (ROBINS‐I)
Petursdottir and Svavarsdottir (2019) Iceland	To evaluate the effectiveness of a therapeutic conversation intervention (FAM‐SOTC) among family caregivers of a person with advanced cancer at home.	Quasi‐experimental study	The FAM‐SOTC intervention consists of two main phases, assessment and intervention, carried out through a therapeutic conversation between a nurse and the caregiver.	—	– Ice‐Family Perceived Support Questionnaire (ICE‐FPSQ). – Depression Anxiety Stress Scale (DASS). – Brief Assessment Scale for Caregivers of the Medically III (BASC).	– Perceived support: *F* = 51.24, *p* < 0.001, *η* ^2^ = 0.522. – Stress at T3 *F* = 4.28, *p* = 0.029, *η* ^2^ = 0.084. – Depression: *F* = 1.77, *p* = 0.183, η^2^ = 0.036 – Anxiety: *F* = 2.30, *p* = 0.120, η^2^ = 0.047 – Burden of care: *F* = 4.67, *p* = 0.012, η^2^ = 0.090 – Negative personal impact: *F* = 4.88, *p* = 0.010, η^2^ = 0.094.	Moderate (ROBINS‐I)
Piamjariyakul et al. (2024) USA	To test whether the family heart failure (HF) palliative and end‐of‐life care intervention (FamPALcare) improved patient and caregiver outcomes.	RCT	FamPALcare (telephone coaching to prepare patients and caregivers with practical skills for specific HF palliative and end‐of‐life care) and usual care.	Usual care (educational materials given to them at the outpatient clinics or upon hospital discharge planning).	– Zarit Caregiver Burden Interview (ZBI). – Patient Health Questionnaire (PHQ‐4).	– Caregiver burden: At 3 months: *M* (SD) 9.48 (4.23) versus 13.38 (7.02); *t* = 2.06, *p* = 0.025 At 6 months: 8.12 (3.67) versus 9.9 3 (7.16); *t* = 0.97, *p* = 0.17 – Depression/Anxiety: At 3 months: M(SD) 2.06 (1.64) vs. 4.53 (3.64); *t* = 2.67, *p* = 0.007 At 6 months: 2.69 (2.01) vs. 4.32 (3.80); *t* = 1.64, *p* = 0.06	Some concerns (RoB 2)
Rochmawati and Saun (2022) Indonesia	To determine the effectiveness of palliative care training for family caregivers (HBPC‐FC) on caregiving readiness and work overload.	Quasi‐experimental study	Nurses give individual training sessions for 3 weeks.	They do not receive training on preparedness to care and work overload.	– Caregiving Inventory (CGI) – Zarit Burden Index (ZBI)	– Caregiving preparedness: improved significantly in the intervention group (pre: 66.53 ± 10.95; post: 131.56 ± 29.55) compared to control (post: 71.20 ± 9.51); *t* = 9.72, *p* < 0.001 – Caregiving burden: decreased significantly in the intervention group (pre: 79.32 ± 8.97; post: 41.32 ± 15.57) compared to control (post: 79.84 ± 5.78); *t* = 11.59, *p* < 0.001	Moderate (ROBINS‐I)
Valero‐Cantero, Casals, Espinar‐Toledo, Barón‐López, et al. (2023) and Valero‐Cantero, Casals, Espinar‐Toledo, et al. (2023) Spain	To investigate the effects on caregiver caregiving burden an quality of life, of listening to music chosen by family caregivers in the home of terminally ill cancer patients.	RCT	Individualized prerecorded music in daily 30‐min sessions for seven consecutive days on a personal technological device; and conventional health care (including basic therapeutic education for palliative care by employing a technological device).	Conventional health care, including basic therapeutic education for palliative care (using a technological device).	– Caregiver Strain Index (CSI) – Quality of Life Family Version (QOL‐FV) – European Quality of Life—five dimensions (EuroQoL‐5D‐5L)	– Caregiver burden: 6.24 (3.30) vs. 7.51 (2.80), (*p* = 0.003) – Quality of life: QOL‐FV: +0.16 (0.75) vs. −0.35 (0.94), *p* = 0.008 EuroQoL‐5D‐5L: +6.29 (10.71) vs. −3.76 (7.26), *p* < 0.001	Low (RoB 2)
von Heymann‐Horan et al. (2019) and von Heymann‐Horan et al. (2023) Denmark	To investigate whether Specialized Palliative Care (SPC) and a dyadic psychological intervention increase stress communication and common coping; and reduce caregiver burden (The Domus RCT).	RCT	Home care by a multidisciplinary team (SPC). Two dyadic sessions+in‐person needs‐based individual/dyadic sessions.	Usual care	– Symptom Checklist‐92 anxiety and depression subscales. – Relationship Ladder. – Dyadic Coping Inventory (DCI) – 12‐item Zarit Burden Interview (ZBI)	– Stress communication: 0.66 (95% CI: −0.04, 1.36); *p* = 0.0142 – Common coping: 0.4 (95% CI: −0.1, 0.9); *p* = 0.0833 – Caregiver burden: Overall: 95% CI (−0.98, 1.67); *p* = 0.5046 Personal strain subscale: 95% CI (−0.55, 1.57); *p* = 0.5623 Role strain subscale: 95% CI (−0.82, 0.29); *p* = 0.2268	Some concerns (RoB 2)

Abbreviations: *d*, Cohen's *d*; *F*, the *F* value comes from ANOVA and indicates whether differences between groups are statistically significant; *p*, *p* value; *Q*, question; SD, standard deviation; *W*, Wilcoxon test statistic; *η* ^2^, partial eta squared.

### 2.4 Risk of Bias Assessment

Methodological quality was determined using the *Cochrane Risk of Bias 2* (RoB 2) tool (Sterne et al. 2019) for randomized clinical trials and the *Risk of Bias In Nonrandomized Studies of Interventions* (ROBINS‐I) tool (Sterne et al. 2016) for nonrandomized studies. Based on the assessment of each item, the overall risk of bias in a study was evaluated as low risk of bias, some concerns, or high risk of bias (Sterne et al. 2019); and as low, moderate, serious, critical risk of bias, or no information (Sterne et al. 2016), respectively. The selection, review, and quality assessment of the studies were independently performed by two authors (LSG and JMM), both of whom had prior training and experience in using the assessment tools. Each author performed the assessments separately and blinded to the other's evaluations. The results were then compared, and any discrepancies were discussed and resolved through consensus. A third author (MOL), who received specific training, also participated in the process under supervision.

## 3 Results

### 3.1 Study Selection

Initially, 2231 articles were obtained from the five databases reviewed. After the identification of duplicate articles, 1114 studies were discarded. Next, 1072 articles were eliminated by reading the title and abstract. After a complete and exhaustive reading of the remaining 45 articles, 28 additional articles were discarded. Thus, 16 studies were selected for analysis of the results (see Figure 2).

### 3.2 Study Characteristics

The characteristics of the 16 included studies, including the research design, subjects, conceptual framework, type of intervention, and measurement tools, are shown in Table 2. Five (31.3%) studies were conducted in the USA, three (18.75%) in Denmark, two (12.5%) in Spain, and one each in Canada, China, Japan, Sweden, Iceland, and Indonesia.

The sample included nine randomized clinical studies (56.3%) (Dionne‐Odom et al. 2022; Gregory and Gellis 2020; Lund et al. 2020; Mooney et al. 2023; Piamjariyakul et al. 2024; Valero‐Cantero, Casals, Espinar‐Toledo, Barón‐López, et al. 2023; Valero‐Cantero, Casals, Espinar‐Toledo, et al. 2023; von Heymann‐Horan et al. 2019; von Heymann‐Horan et al. 2023). Among these randomized studies, one was a cluster study (6.25%) (Lund et al. 2020) and two were pilot studies (12.5%) (Dionne‐Odom et al. 2022; Gregory and Gellis 2020). In addition to these, seven quasi‐experimental studies (43.8%) were also included (Boyko et al. 2021; Chow et al. 2024; Fleisher et al. 2023; Ito and Tadaka 2022; Norinder et al. 2024; Petursdottir and Svavarsdottir 2019; Rochmawati and Saun 2022). Fourteen interventions were identified from these 16 original studies.

Overall, the 14 interventions included a sample of 1922 participants with a mean age of 58.7 years old. Of the total participants, 893 identified themselves as the patient's spouse (46.5%), and 502 as the adult child (26.1%). Furthermore, in 12 of the 14 interventions analyzed, the caregivers were predominantly women (85.7%).

### 3.3 Risk of Bias

The risk of bias of the studies is presented in Table 2 (see Appendix B of the Supporting Information for more detailed information). Among the nine randomized clinical studies, two studies showed a low risk of bias (Valero‐Cantero, Casals, Espinar‐Toledo, Barón‐López, et al. 2023; Valero‐Cantero, Casals, Espinar‐Toledo, et al. 2023); and the remaining seven were evaluated as having some concerns (Dionne‐Odom et al. 2022; Gregory and Gellis 2020; Lund et al. 2020; Mooney et al. 2023; Piamjariyakul et al. 2024; von Heymann‐Horan et al. 2019; von Heymann‐Horan et al. 2023). These risks of bias are largely due to issues such as lack of participant blinding and incomplete reporting of randomization processes (Dionne‐Odom et al. 2022; Gregory and Gellis 2020; Mooney et al. 2023; von Heymann‐Horan et al. 2019; von Heymann‐Horan et al. 2023). Handling of missing data varied across studies. Some managed losses effectively through intention‐to‐treat analyses (Gregory and Gellis 2020), while others reported higher attrition rates due to patient mortality or caregiver dropout, which increased the risk of bias related to missing outcome data (Dionne‐Odom et al. 2022; Lund et al. 2020; Piamjariyakul et al. 2024; von Heymann‐Horan et al. 2019; von Heymann‐Horan et al. 2023). Despite this, most used validated outcome measures and transparent reporting, which reduced the risk of selective reporting bias.

Among the quasi‐experimental studies, with the exception of the study by Fleisher et al. (2023), which had a serious risk of bias, the remaining six showed a moderate risk of bias (Boyko et al. 2021; Chow et al. 2024; Ito and Tadaka 2022; Norinder et al. 2024; Petursdottir and Svavarsdottir 2019; Rochmawati and Saun 2022). These risk‐of‐bias results are mainly due to inadequate control of confounding variables and non‐random or voluntary selection of participants, which may have resulted in unrepresentative samples (Boyko et al. 2021; Chow et al. 2024; Ito and Tadaka 2022; Fleisher et al. 2023). In addition, although the classification and application of interventions were generally consistent, in some cases variations may have affected the results (Chow et al. 2024). Another key factor was the treatment of missing data, as high dropout rates or insufficient analysis of incomplete data influenced the interpretation of results (Fleisher et al. 2023; Norinder et al. 2024). Finally, the use of self‐reports and lack of blinding increased the risk of bias in outcome measurement (Chow et al. 2024; Rochmawati and Saun 2022).

Notably, a meta‐analysis of the results could not be performed due to substantial heterogeneity across the included studies. As outlined in the Cochrane Handbook for Systematic Reviews of Interventions (Deeks et al. 2024), narrative synthesis is recommended when variations in populations, interventions, outcomes, or study designs make statistical pooling uninformative or potentially misleading. This was precisely the case of this review: interventions varied widely in components, duration, intensity, delivery methods, and theoretical underpinnings; outcomes were assessed using diverse instruments at different time points; and the study populations included caregivers of individuals with a range of chronic and progressive conditions, each with distinct trajectories and care needs.

### 3.4 Synthesis of the Results

#### 3.4.1 Theoretical Framework for Interventions

The methodological frameworks that guided the interventions retrieved were mentioned in 10 interventions (71.4%): the Integrated Community End‐of‐Life Care Support Team (ICEST) model (Chow et al. 2024); the Pearling Stress Model (Dionne‐Odom et al. 2022); the Nezu, Nezu, and D'Zurilla Five‐step model (Gregory and Gellis 2020); emotional competence (Ito and Tadaka 2022); Carer Support Needs Assessment Tool (CSNAT) underpinned by a person‐centred framework (Lund et al. 2020; Norinder et al. 2024); the Calgary Family Assessment (CFAM) and Calgary Family Intervention (CFIM) models (Petursdottir and Svavarsdottir 2019); the Coaching model for heart failure home care (Piamjariyakul et al. 2024); the Andershed & Ternestedt model (Rochmawati and Saun 2022) and the Existential–Phenomenological Therapy (EPT) (von Heymann‐Horan et al. 2019; von Heymann‐Horan et al. 2023).

#### 3.4.2 Mode of Delivery

Fourteen interventions were identified from the 16 primary studies. Seven were exclusively face‐to‐face interventions (Chow et al. 2024; Gregory and Gellis 2020; Lund et al. 2020; Norinder et al. 2024; Petursdottir and Svavarsdottir 2019; Rochmawati and Saun 2022; von Heymann‐Horan et al. 2019; von Heymann‐Horan et al. 2023); two were online (14.3%) (Boyko et al. 2021; Ito and Tadaka 2022); one was delivered via telephone (7.1%) (Piamjariyakul et al. 2024); and one used an electronic device (7.1%) (Valero‐Cantero, Casals, Espinar‐Toledo, Barón‐López, et al. 2023; Valero‐Cantero, Casals, Espinar‐Toledo, et al. 2023). The remaining three (21.4%) employed a combined modality: Dionne‐Odom et al. (2022) integrated telephone‐delivered coaching, follow‐up calls, and written materials; Mooney et al. (2023) combined online symptom self‐reporting with daily automated interactive voice response telephone coaching tailored to symptom severity; and Fleisher et al. (2023) incorporated home visits with written resources, mentoring calls from other caregivers, and telehealth (Fleisher et al. 2023) (See Table 3 for further details).

**TABLE 3:** Characteristics of the interventions.

Author (Year) Country	Setting	Recipients (*N* = participants)	Providers: role	Modality	Duration	Frequency
Boyko et al. (2021) Canada	Home	Caregivers (*N* = 176)	Self‐administered	Online (web‐based course)	Free	Seven modules 1 h each
Chow et al. (2024) China	Home	Caregivers (*N* = 251)	Doctors, nurses, social workers, personal care workers and volunteers: providing caregiver empowerment and support through education, counseling, training, and facilitated family interactions.	Face‐to‐face	3 months	At least two interviews
Dionne‐Odom et al. (2022) USA	Home	Caregivers (*N* = 63)	Specially‐trained palliative care lay navigators: delivered one‐on‐one coaching sessions via telephone to caregivers, offering educational and emotional support.	Combined modality: telephone‐delivered coaching, telephonic follow‐up, and written materials (Cornerstone Family Toolkit)	24 weeks	6 weekly sessions (one per week), each lasting 20–60 min (average: 32 min). Followed by monthly check‐in calls through Week 24.
Fleisher et al. (2023) USA	Home	Patient‐caregiver dyad (*N* = 65)	Nurse, social worker, Parkinson's specialist: home visits focused on symptom management, medication, safety and psychological needs. Parkinson's Disease family caregivers: peer mentors providing weekly support and coaching calls.	Combined modality: in‐person visits, telehealth support, telephone‐based peer mentoring, and written materials	12 months	Home visits: 4 total (Months 1, 4, 8, and 12; timing not specified). Peer mentoring 1 phone call per week for 16 weeks (between visits 2 and 3).
Gregory and Gellis (2020) USA	Home	Caregiver (*N* = 37)	Clinical social worker with hospice experience: delivered the PST‐Hospice intervention, and conducted initial interviews.	Face‐to‐face	5 weeks	One session per week, for a total of five sessions (average: 45 min)
Ito and Tadaka (2022) Japan	Home	Caregivers (*N* = 60)	Self‐administered	Online (daily diary writing)	7 days	Once per day for seven consecutive days
Lund et al. (2020) Denmark	Outpatient clinic Home	Caregivers (*N* = 437)	Interdisciplinary teams (specialized nurses and medical doctors, social workers, physiotherapists, psychologists and priests): intervention was delivered by nurses and additional professionals in some teams.	Face‐to‐face (Telephone and video allowed when necessary)	1 month	First session: within 13 days of enrollment, lasting an average of 43 min (range: 10–90 min) Second session: 15–27 days after enrollment, lasting an average of 32 min (range 10–70 min)
Mooney et al. (2023) USA	Home	Caregivers (*N* = 332)	Self‐administered; hospice nurses received automated alerts for moderate‐to‐severe symptoms and responded as needed (visit, call, or monitor)	Combined modality: online self‐reporting of symptoms plus daily automated interactive voice response telephone coaching tailored by symptom severity algorithms	From hospice admission until patient death; mean duration was 37 days, median 18 days.	Daily symptom reporting; average call duration 11 min for intervention group versus 9 min in control.
Norinder et al. (2024) Sweden	Home	Caregivers (*N* = 33)	Specialized palliative care nurses: facilitated the intervention	Face‐to‐face	Approximately 5 weeks	Not explicitly stated; likely at least two sessions (initial plus follow‐up) with ongoing reviews
Petursdottir and Svavarsdottir (2019) Iceland	Home	Caregivers (*N* = 48)	Advanced palliative care nurses: assessed family dynamics and facilitated therapeutic conversations focused on support, strengths and coping	Face‐to‐face	4 weeks	Two sessions were delivered, each lasting 60–90 min, held once per week over two consecutive weeks.
Piamjariyakul et al. (2024) USA	Home	Patient‐caregiver dyad (*N* = 39)	Nurse clinician: coached patients and their family caregivers on heart failure palliative symptom management and guided advanced directives discussions.	Telephone calls	3‐month intervention with a 6 months follow‐up	Five structured telephone coaching sessions, each lasting 60–90 min.
Rochmawati and Saun (2022) Indonesia	Home	Caregivers (*N* = 50)	Postgraduate students in nursing: to deliver structured training in symptom management and caregiving skills.	Face‐to‐face	3 weeks	One session per week, each lasting 1–2 h.
Valero‐Cantero, Casals, Espinar‐Toledo, Barón‐López, et al. (2023) and Valero‐Cantero, Casals, Espinar‐Toledo, et al. (2023) Spain	Home	Caregivers (*N* = 82)	Self‐administered	Asynchronous delivery via mp3 player or mobile phone	7 days	One session per day, lasting 30 min each
von Heymann‐Horan et al. (2019) and von Heymann‐Horan et al. (2023) Denmark	Home	Patient‐caregiver dyad (*N* = 249)	Psychologists: delivered the dyadic psychological intervention based on existential‐phenomenological therapy, conducted needs assessments, and provided therapeutic support. SPC team members, (district nurses, and, when possible, the general practitioner): participated in the initial home‐care conference to coordinate care.	Face‐to‐face (telephone allowed when necessary)	6‐month intervention or until early bereavement	Two dyadic sessions initiated the intervention, followed by monthly telephone needs assessments and needs‐based sessions, which lasted up to 90 min.

#### 3.4.3 Duration of Interventions

The durations of the interventions included ranged from 1 week (Ito and Tadaka 2022; Valero‐Cantero, Casals, Espinar‐Toledo, Barón‐López, et al. 2023; Valero‐Cantero, Casals, Espinar‐Toledo, et al. 2023) to twelve months (Fleisher et al. 2023). Nine interventions lasted less than three months (50%) (Boyko et al. 2021; Chow et al. 2024; Gregory and Gellis 2020; Lund et al. 2020; Mooney et al. 2023; Norinder et al. 2024; Petursdottir and Svavarsdottir 2019; Piamjariyakul et al. 2024; Rochmawati and Saun 2022), while two interventions lasted six months (28.6%) (Dionne‐Odom et al. 2022; von Heymann‐Horan et al. 2019; von Heymann‐Horan et al. 2023).

The number of sessions between patients/caregivers and providers ranged from two (Chow et al. 2024; Lund et al. 2020; Norinder et al. 2024; Petursdottir and Svavarsdottir 2019) to seven sessions (Ito and Tadaka 2022; Valero‐Cantero, Casals, Espinar‐Toledo, Barón‐López, et al. 2023; Valero‐Cantero, Casals, Espinar‐Toledo, et al. 2023) and between 5 (Piamjariyakul et al. 2024) and more than 15 telephone calls (Fleisher et al. 2023; Mooney et al. 2023). The duration of the interventions ranged from 20 min (Dionne‐Odom et al. 2022) to 120 min (Rochmawati and Saun 2022) (See Table 3 for further details).

#### 3.4.4 Intervention Providers

Two interventions (14.3%) were led by palliative care nurses (Norinder et al. 2024; Petursdottir and Svavarsdottir 2019); one (7.1%) by postgraduate nurses (Rochmawati and Saun 2022); one (7.1%) by specially trained lay people (Dionne‐Odom et al. 2022); one (7.1%) by psychologists (von Heymann‐Horan et al. 2019; von Heymann‐Horan et al. 2023); one (7.1%) by social workers (Gregory and Gellis 2020); and three (21.4%) by a multidisciplinary team. One of these teams consisted of a nurse, a social worker, a physician, and peer mentors (Fleisher et al. 2023); another consisted of nurses, physicians, social workers, psychologists, physiotherapists, and priests (Lund et al. 2020); and the other team included nurses, physicians, social workers, home care assistants, and volunteers (Chow et al. 2024).

On the other hand, three interventions (21.4%) were self‐administered by the participants, although they were designed by the research team (Ito and Tadaka 2022; Mooney et al. 2023; Boyko et al. 2021), with one of them making use of artificial intelligence (Mooney et al. 2023). Additionally, in one of the interventions (7.1%), participants self‐administered the intervention and selected the music themselves (Valero‐Cantero, Casals, Espinar‐Toledo, Barón‐López, et al. 2023; Valero‐Cantero, Casals, Espinar‐Toledo, et al. 2023). In the studies by Lund et al. (2020) and Piamjariyakul et al. (2024), the participants collaborated in adapting predesigned interventions according to their needs (See Table 3 for further details).

#### 3.4.5 Intervention Recipients

Eleven interventions (78.6%) focused solely on primary caregivers (Boyko et al. 2021; Chow et al. 2024; Dionne‐Odom et al. 2022; Gregory and Gellis 2020; Ito and Tadaka 2022; Lund et al. 2020; Mooney et al. 2023; Norinder et al. 2024; Petursdottir and Svavarsdottir 2019; Rochmawati and Saun 2022; Valero‐Cantero, Casals, Espinar‐Toledo, Barón‐López, et al. 2023; Valero‐Cantero, Casals, Espinar‐Toledo, et al. 2023). In comparison, three interventions (21.4%) included a dyad of patient and family member (Fleisher et al. 2023; Piamjariyakul et al. 2024; von Heymann‐Horan et al. 2019; von Heymann‐Horan et al. 2023) (see Table 3 for further details).

The pathologies of ill family members were cancer in seven interventions (50%) (Dionne‐Odom et al. 2022; Ito and Tadaka 2022; Lund et al. 2020; Mooney et al. 2023; Petursdottir and Svavarsdottir 2019; Valero‐Cantero, Casals, Espinar‐Toledo, Barón‐López, et al. 2023; Valero‐Cantero, Casals, Espinar‐Toledo, et al. 2023; von Heymann‐Horan et al. 2019; von Heymann‐Horan et al. 2023); Parkinson's disease in another (7.1%) (Fleisher et al. 2023); heterogeneous illnesses (oncology and other pathologies such as central nervous system disorders, heart failure, stroke, diabetes, or chronic obstructive pulmonary disease) were reported in three interventions (21.4%) (Gregory and Gellis 2020; Norinder et al. 2024; Rochmawati and Saun 2022); and patients receiving palliative home care for a variety of situations were found in another two interventions (14.3%) (Boyko et al. 2021; Chow et al. 2024).

#### 3.4.6 Intervention Domains

After the interventions were integrated and analyzed, three recurrent domains were identified: psychoeducational, practical, and behavioral support.

*Psychoeducational support domain*. This domain focuses on providing information and training that enhance caregivers' understanding and skills related to managing stress, emotional coping, and the burden of care. For instance, Boyko et al. (2021) implemented an online program aimed at improving non‐professional caregivers' competencies in various aspects of care, ranging from physical to emotional and spiritual support. Similarly, Gregory and Gellis (2020) utilized psychoeducational interventions to reduce caregivers' anxiety and depression through problem‐solving therapies, while Mooney et al. (2023) provided remote educational coaching to improve caregivers' capacity to handle both emotional and practical responsibilities in home care settings. In total, 11 interventions (78.6%) included this domain, reflecting a common trend towards enhancing caregivers' cognitive and emotional skills, equipping them with essential knowledge to face the challenges of care (Boyko et al. 2021; Dionne‐Odom et al. 2022; Fleisher et al. 2023; Gregory and Gellis 2020; Lund et al. 2020; Mooney et al. 2023; Norinder et al. 2024; Petursdottir and Svavarsdottir 2019; Piamjariyakul et al. 2024; Rochmawati and Saun 2022; von Heymann‐Horan et al. 2019; von Heymann‐Horan et al. 2023).

*Practical support domain*. This domain focuses on providing caregivers with tangible tools and strategies to effectively manage the physical and logistical demands of home care. This domain is clearly visible in studies such as Chow et al. (2024), Lund et al. (2020) and Norinder et al. (2024), where interventions provided support in care planning and organizing the home environment to improve caregivers' quality of life. In other cases, providers offered referrals to community services available at the territorial and state level or the possibility of loaning medical equipment (Dionne‐Odom et al. 2022; Fleisher et al. 2023). In the end, five of the interventions reviewed (35.7%) included this domain, underscoring its importance in managing the many tasks involved in the ongoing care of their family member with advanced illness (Dionne‐Odom et al. 2022; Chow et al. 2024; Lund et al. 2020; Norinder et al. 2024; Piamjariyakul et al. 2024).

*Behavioral Support Domain*. This domain was addressed in three of the interventions examined (21.4%) (Petursdottir and Svavarsdottir 2019; Ito and Tadaka 2022; Valero‐Cantero, Casals, Espinar‐Toledo, Barón‐López, et al. 2023; Valero‐Cantero, Casals, Espinar‐Toledo, et al. 2023). These activities were intended to provide caregivers with strategies to improve their attitudes, behaviors, and emotions related to their caregiving experience. Most of the interventions focused on improving caregivers' coping skills through individual reflective activities and the use of daily diaries (Ito and Tadaka 2022) or through activities involving taking time out of their day to listen to music chosen personally by them (Valero‐Cantero, Casals, Espinar‐Toledo, Barón‐López, et al. 2023; Valero‐Cantero, Casals, Espinar‐Toledo, et al. 2023).

#### 3.4.7 Effects of the Interventions

Due to the heterogeneity of the outcome measures used in the studies reviewed, it was not possible to determine which intervention was the most effective in addressing the needs of the caregivers under study. However, some statistically significant results of the interventions were identified. Table 4 includes a graphical summary of the effectiveness of the interventions in each study.

**TABLE 4:** Effect of the interventions.

	Depression	Anxiety	Distress	Quality of life	Care burden	Self‐efficacy/competence to care	Perceived support	Intimacy	Care planning	Problem‐solving ability	Quality of information received	Mood and vitality	Ability to cope with the situation (stress)
Boyko et al. (2021)						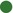							
Chow et al. (2024)	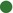				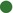		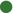	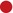	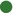		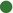		
Dionne‐Odom et al. (2022)		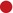	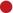	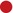									
Fleisher et al. (2023)	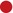	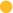			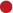	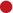							
Gregory and Gellis (2020)	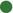			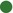						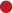			
Ito and Tadaka (2022)	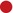			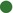									
Lund et al. (2020)			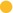	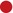	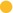						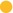		
Mooney et al. (2023)												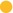	
Norinder et al. (2024)				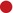	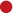	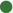							
Petursdottir and Svavarsdottir (2019)	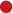	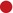			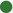		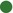						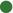
Piamjariyakul et al. (2024)	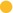	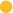			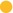								
Rochmawati and Saun (2022)					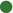	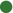							
Valero‐Cantero, Casals, Espinar‐Toledo, Barón‐López, et al. (2023) and Valero‐Cantero, Casals, Espinar‐Toledo, et al. (2023)				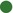	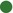								
von Heymann‐Horan et al. (2019) and von Heymann‐Horan et al. (2023)					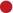								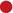

*Note:* White, not measured; Green, statistical superiority favoring intervention; Yellow, statistical superiority favoring intervention only at some measurement time; Red, no statistical superiority favoring intervention.

The following is a summary of the six outcomes most frequently measured in the reviewed studies: depression, anxiety, distress, quality of life, caregiving burden, and self‐efficacy/competence to care. All the studies evaluated the interventions at multiple time points, and the follow‐up period ranged from 1 week (Ito and Tadaka 2022; Valero‐Cantero, Casals, Espinar‐Toledo, Barón‐López, et al. 2023; Valero‐Cantero, Casals, Espinar‐Toledo, et al. 2023) to 24 months (Fleisher et al. 2023), with a follow‐up of four to eight weeks being the most common (Gregory and Gellis 2020; Lund et al. 2020; Mooney et al. 2023; Norinder et al. 2024; Petursdottir and Svavarsdottir 2019; Rochmawati and Saun 2022).

*Depression*. Depression was measured in six of the 14 included interventions. Of these, only two (22.3%) reported positive effects of the interventions (Chow et al. 2024; Gregory and Gellis 2020). In the case of the intervention developed by Piamjariyakul et al. (2024), the intervention demonstrated effectiveness on depression at 3 months (*t* = 2.67, *p* = 0.007) but was not sustained at 6 months (*t* = 1.64, *p* = 0.06). For the remaining interventions, there were no statistically significant changes in depression during all their study periods (Fleisher et al. 2023; Ito and Tadaka 2022; Petursdottir and Svavarsdottir 2019).

*Anxiety*. On the other hand, anxiety was measured in four of the included interventions (28.6%) (Dionne‐Odom et al. 2022; Fleisher et al. 2023; Petursdottir and Svavarsdottir 2019; Piamjariyakul et al. 2024). However, positive effects of the interventions on this parameter were found in only one of these interventions, although they were not statistically significant [−1.94 (95% CI: −0.98 to 0.11); *d* = −0.44] (Dionne‐Odom et al. 2022). Fleisher et al. (2023) showed a slight momentary improvement between home visits 1 and 4 that was statistically significant [7.07 (4.1) to 5.96 (3.95); *p* = 0.049]; and Piamjariyakul et al. (2024) showed a significant effect at 3 months [*t* = 2.67, *p* = 0.007], although it was not sustained over time (at 6 months *t* = 1.64, *p* = 0.06).

*Distress*. Finally, distress was measured in two of the interventions included in this study (14.3%) (Dionne‐Odom et al. 2022; Lund et al. 2020). In the study by Lund et al. (2020), a positive effect on distress was found at the first assessment (2 weeks after the start of the intervention) (*d* = 0.12; *p* = 0.0178), but this effect was not sustained over time (*d* = 0.04; *p* = 0.4424). In comparison, the study by Dionne‐Odom et al. (2022) reported a positive effect of the intervention at subsequent follow‐up regarding this parameter, despite the fact that it did not reach statistical significance [−2.29 (−0.88 a 0.24); *d* = −0.32].

The instrument most commonly used to measure depression, anxiety, and distress was the 14‐item Hospital Anxiety and Depression Scale (HADS) (Dionne‐Odom et al. 2022; Fleisher et al. 2023) (see Table 2 for the remaining instruments used).

*Quality of life*. Quality of life was assessed in six interventions (42.9%), with four demonstrating positive effects on this outcome (Dionne‐Odom et al. 2022; Gregory and Gellis 2020; Ito and Tadaka 2022; Valero‐Cantero, Casals, Espinar‐Toledo, et al. 2023). However, the effect reported by Dionne‐Odom et al. (2022) was not statistically significant [−1.56 (95% CI: −6.23 to 3.12); *d* = −0.07]. In both studies that did not report positive effects, the CSNAT‐I intervention was used: Lund et al. (2020) found no significant effect on quality of life as measured by the EORTC QLQ‐C30 (at 2 weeks: *d* = 0.03, *p* = 0.9274; at 4 weeks: *d* = 0.11, *p* = 0.7534), and Norinder et al. (2024) also found no statistically significant changes in the QOLLTI‐F at either time point [Mdn = 6.0 (5.0–8.0) to 6.0 (5.0–8.0), *p* = 0.645].

The most used measurement instrument for this outcome was the Caregiver Quality of Life Index–Cancer (CQOLC) (see Table 2).

*Caregiver burden*. Ten interventions addressed caregiver burden (71.4%) (Chow et al. 2024; Fleisher et al. 2023; Lund et al. 2020; Mooney et al. 2023; Norinder et al. 2024; Petursdottir and Svavarsdottir 2019; Piamjariyakul et al. 2024; Rochmawati and Saun 2022; Valero‐Cantero, Casals, Espinar‐Toledo, Barón‐López, et al. 2023; von Heymann‐Horan et al. 2023). Of these, five interventions yielded positive results according to this outcome (Chow et al. 2024; Mooney et al. 2023; Petursdottir and Svavarsdottir 2019; Rochmawati and Saun 2022; Valero‐Cantero, Casals, Espinar‐Toledo, Barón‐López, et al. 2023). The study conducted by Piamjariyakul et al. (2024) showed a significant effect at three months (*t* = 2.06, *p* = 0.025), although at 6 months follow‐up it was no longer significant (*t* = 0.97, *p* = 0.17); similar to what occurred in the study of Lund et al. (2020) (T1: *d* = 0.15; *p* = 0.1875; T2: *d* = 0.19; *p* = 0.1533). On the other hand, Fleisher et al. (2023) highlighted that although caregiver burden did not decrease after the intervention, the intervention group maintained a constant burden throughout the study, in contrast to the increase in caregiver burden in the control group.

Numerous measurement instruments were used in the studies (see Table 2); only the Zarit Burden Index scale was used at least twice (Rochmawati and Saun 2022; von Heymann‐Horan et al. 2023).

*Self‐efficacy and competency to care*. Although self‐efficacy and perceived competence are often considered differently, the reviewed studies used both concepts interchangeably to refer to the perception of one's ability to cope with stressful situations related to caregiving roles. Therefore, in the results of this review, the two concepts are considered the same. Four studies (28.6%) (Boyko et al. 2021; Fleisher et al. 2023; Norinder et al. 2024; Rochmawati and Saun 2022) measured self‐efficacy/competence in providing care. However, only three reported a statistically significant improvement in this outcome (Boyko et al. 2021; Norinder et al. 2024; Rochmawati and Saun 2022), especially in terms of educational support and perceived readiness to care for loved ones.

Numerous measurement instruments were used in the present study (see Table 2), including the Caregiver Self‐Efficacy Scale (CSES) (Fleisher et al. 2023) and the Caregiving Inventory (CGI) (Rochmawati and Saun 2022).

## 4 Discussion

The main objective of this review was to identify the impact of home interventions on family members serving as caregivers of people with advanced illness. The secondary objective was to analyze the characteristics and components of these interventions. This discussion will focus on the most relevant and novel aspects from the results to guide the design and implementation of future interventions that address caregivers' needs.

It has been reported in various studies that distress and depression are recurrent issues among caregivers (Holm et al. 2020; Kozlov et al. 2019). The interventions included in this review focused on caregivers' psychological needs, though the underlying causes of depression and distress are not individually addressed, increasing the likelihood of relapse. This may explain why a positive effect on this domain has not been observed in several interventions (Fleisher et al. 2023; Ito and Tadaka 2022; Petursdottir and Svavarsdottir 2019; von Heymann‐Horan et al. 2019).

Similarly, it should be noted that the new caregiving role acquired by these family members directly impacts their quality of life (Alam et al. 2020). The interventions analyzed address this domain from different perspectives: self‐care Valero‐Cantero, Casals, Espinar‐Toledo, Barón‐López, et al. (2023; Valero‐Cantero, Casals, Espinar‐Toledo, et al. 2023), help‐seeking, organization, and future planning (Dionne‐Odom et al. 2022); emotional support via journals (Ito and Tadaka 2022); problem‐solving therapy (Gregory and Gellis 2020); and tools identifying needs for health care professionals to address (Lund et al. 2020; Norinder et al. 2024). The diversity of approaches and results highlights the complexity of the concept of quality of life, which varies within and across disciplines (Haraldstad et al. 2019). According to Teoli and Bhardwaj (2023), quality of life encompasses health (physical, mental and spiritual), social relations, education level, work environment, social status, wealth, sense of security and safety, freedom, autonomy in decision‐making, and physical environment. The World Health Organization (WHO) (2024) adds that an individual's perception of quality of life is influenced by the individual's goals, culture, expectations, norms, and concerns; meaning that static interventions are insufficient—interventions must consider each individual's uniqueness.

Caregiver burden and caregiving self‐efficacy/competence must be addressed in a personalized manner. Caregivers are diverse, so research should clarify similarities and differences to tailor supportive policies and programs (Utz and Warner 2022). The meanings, roles, and dynamics of relationships differ; for example, spouses may be motivated by the romantic vow “in sickness and in health”, while child caregivers may be motivated by a sense of reciprocal duty or filial obligation (Berg et al. 2021). Children often feel less prepared, with caregiving interfering with their life milestones, such as education, forming intimate relationships, creating and raising a family, and achieving financial stability (Too et al. 2023; Waters et al. 2021).

Similarly, the terms “self‐efficacy” and “competence” in caring should not be used interchangeably. Khan et al. (2021) define self‐efficacy as confidence in managing caregiving stressors, learning medical skills, controlling disruptive thoughts, accessing community support, assisting with activities of daily living, and maintaining a good relationship with his/her social environment. On the other hand, competence refers not only to the caregiver's feeling of being able to manage the provision of care (Vernooij‐Dassen et al. 1997) but also to the results of care obtained for the patient (Stansfeld et al. 2019). Therefore, researchers must consider these differences when designing interventions for caregivers.

As Utz and Warner (2022) noted in their study, the most effective interventions are those that consider family caregivers' diverse experiences and circumstances, regardless of their age or relationship with the person they care for. This assertion is consistent with those in other studies, such as Martín‐Martín et al. (2022) and Vermorgen et al. (2021), in which caregivers were considered recipients of care because their well‐being was considered not only during the loved one's illness but also after the death of the loved one. Therefore, perhaps one of the determining aspects of the success of these interventions would be the family's involvement in the design of the interventions intended to care for them. However, the need to adopt a family perspective should be emphasized. The whole family suffers irremediably from the situation that their loved one is experiencing, but at the same time, they try to care for and support the loved one. The disease significantly impacts all family members, and in one way or another, they modify their roles in the family unit (Lee and Yun 2018; Martín‐Martín et al. 2022). For all these reasons, the whole family faces a challenge and a crisis that tests its stability. Although some studies included in this review attempted to adapt their interventions to the individual needs arising from caregiving, they continued to include exclusively the primary caregiver; and, moreover, the exchange of ideas was embedded within already predesigned interventions (Lund et al. 2020). These may be some of the reasons why no positive effects of these interventions were found.

Regarding the providers of the interventions included in this review, it was observed that in only three of the interventions analyzed (21.4%) a multidisciplinary team was in charge of administering the intervention (Chow et al. 2024; Fleisher et al. 2023; Lund et al. 2020). Although a minority of studies included this type of team, a study by Fernando and Hughes (2019) stated that a multidisciplinary team is best suited to provide end‐of‐life care. Nevertheless, the present systematic review has shown that multidisciplinary teams have disparate effects on the intervened domains, while interventions led by a single profession have generally obtained the most significant benefit on the measured variables: specially trained lay guides (Dionne‐Odom et al. 2022), social workers (Gregory and Gellis 2020), nurses (Petursdottir and Svavarsdottir 2019; Rochmawati and Saun 2022). Therefore, further studies from a multidisciplinary perspective are necessary to determine the potential benefits of this approach.

Notably, the most common type of provider offering home care to these caregivers is nursing professionals. Their presence, either exclusively or in conjunction with other professionals, was a feature of eight interventions (57.1%) (Chow et al. 2024; Fleisher et al. 2023; Lund et al. 2020; Mooney et al. 2023; Norinder et al. 2024; Petursdottir and Svavarsdottir 2019; Piamjariyakul et al. 2024; Rochmawati and Saun 2022). Interventions led or co‐led by nurses generally reported significant improvements in caregiver burden (e.g., Rochmawati and Saun 2022; Piamjariyakul et al. 2024), perceived support (Petursdottir and Svavarsdottir 2019), and preparedness for caregiving (Norinder et al. 2024). In contrast, interventions without nurse involvement—such as those based on digital tools or psychosocial support programs delivered by other professionals (e.g., Boyko et al. 2021; Gregory and Gellis 2020; Valero‐Cantero, Casals, Espinar‐Toledo, Barón‐López, et al. 2023, 2023)—also showed benefits but tended to focus more on emotional aspects such as distress or quality of life, with mixed or modest effects on caregiving competence or burden. These findings highlight the particular value of nurse‐led or nurse‐supported interventions in addressing both emotional and practical caregiving challenges. As Martín‐Martín et al. (2016) emphasized, the essence of nursing lies in the relational and holistic approach to the care of the person and their family. This orientation positions nurses in a privileged role to respond directly and comprehensively to caregivers' needs, while the involvement of other professionals can provide complementary expertise for specific demands (Martín‐Martín et al. 2022).

Despite the insights gained from this review, certain limitations must be acknowledged. The primary limitation is the heterogeneity of the included studies, which made direct comparisons between interventions difficult and hindered the determination of the most effective intervention. Differences in the types of interventions, the contexts in which they were applied, their duration, the outcomes assessed, and the instruments used to measure these outcomes made it impossible to conduct a meta‐analysis. For this reason, the results are predominantly presented in a narrative format, limiting the ability to draw generalizable conclusions about the effectiveness of the interventions.

Moreover, the observed effects of the interventions could be strongly influenced by the cultural context of the countries in which the studies were conducted. Factors such as cultural differences, the educational level of the participants, and the role and significance of family in each region may have affected the outcomes and their generalizability. This cultural variability poses challenges regarding the applicability of the interventions to other contexts or populations. In addition, although gender distribution was reported in several studies, none of them explored differential outcomes by gender, which limits understanding of how caregiving burdens and intervention effects may vary across genders. This represents a gap in the evidence that future studies should address by incorporating gender‐sensitive analyses.

Another notable limitation is the lack of data regarding the long‐term impact of the interventions. Most of the included studies evaluated outcomes over relatively short follow‐up periods (ranging 1–24 weeks), with only one study (Fleisher et al. 2023) extending follow‐up to approximately 1 year. As a result, the sustainability of intervention benefits over time remains uncertain. This represents a significant gap in the literature, particularly given that caregivers' needs and experiences evolve throughout the illness trajectory, including bereavement and long‐term adjustment periods. Without evidence on the persistence of intervention benefits, it is difficult to determine their overall value in supporting caregivers across the continuum of care. Future research should therefore prioritize the inclusion of long‐term follow‐up assessments to capture the enduring effects (or lack thereof) of support interventions. Longitudinal studies could shed light on how caregivers' psychological, emotional, and practical needs change over time, and how interventions might need to be adapted or sustained to meet these evolving demands. Finally, the lack of long‐term outcome data should be acknowledged as a limitation when interpreting the short‐term results presented in this review.

Furthermore, several methodological limitations of the included studies must be acknowledged. In particular, shortcomings related to randomization procedures and blinding may have introduced bias and reduced the reliability of the findings. Most studies also presented a moderate risk of attrition bias, a common challenge in research involving caregivers of patients with advanced chronic illnesses and unpredictable disease trajectories (Cormican et al. 2022). Although we have transparently reported the risk of bias for each study, these limitations inherent to the primary research constrain the overall strength and validity of the conclusions. Future trials should adopt more rigorous designs, incorporating robust randomization, appropriate blinding, and strategies to minimize attrition.

Finally, although not the primary focus of this review, contextual and structural barriers such as financial constraints, digital literacy, and access to professional care were rarely reported in the studies reviewed. These external factors may significantly influence the feasibility, accessibility, and scalability of caregiver interventions, and should be systematically investigated in future research.

## 5 Conclusion

This systematic review was conducted to analyze existing interventions to assist caregivers in caring for loved ones with advanced illness at home. A range of interventions—delivered by different professionals and targeting either caregivers alone or caregiver‐patient dyads—were identified in response to the diverse and complex needs reported by caregivers. Notably, nurses were involved in most of the interventions analyzed, which may reflect their alignment with the holistic and family‐centered philosophy that characterizes nursing practice and their potential role in supporting home‐based caregiving.

However, the overall strength of the evidence is limited. Most studies included in the review showed moderate to high risk of bias, and substantial heterogeneity in intervention formats, durations, and outcome measures hampers direct comparisons and definitive conclusions. Although some interventions demonstrated short‐term benefits, the durability and broader applicability of these effects remain unclear. Future research should therefore prioritize the development and rigorous evaluation of these interventions in diverse healthcare and cultural settings. In the meantime, and while acknowledging the current limitations in the evidence base, efforts to improve support for caregivers may help enhance their experience and, potentially, contribute to enabling individuals with advanced chronic illness to remain at home during the later stages of illness.

## 6 Relevance for Clinical Practice

This review highlights the need to prioritize caregivers as essential participants in the care of people with advanced chronic illness at home. Although nurses were the most frequent providers in the interventions analyzed—reflecting their prominent role in caregiving contexts—the available evidence does not yet allow firm conclusions about which professional profiles or team structures are most effective. In particular, the potential benefits of multidisciplinary teams, though less commonly implemented, warrant further exploration.

Among the approaches identified, personalized, family‐centered interventions—especially those co‐designed with caregivers—emerge as promising strategies to address the varied and evolving needs of those providing care. Strengthening caregiver support in this way may not only enhance their own well‐being, but also help sustain home‐based care for patients, honoring their preferences and supporting continuity across settings. Nevertheless, given the methodological limitations of the current evidence base, these conclusions must be considered preliminary. Clinical implementation should therefore proceed with caution and be accompanied by further high‐quality research.

## Author Contributions

Conceptualization: L.S.G., M.O.L., J.M.M. Methodology: L.S.G., M.O.L., J.M.M. Validation: L.S.G., M.O.L., J.M.M. Formal analysis: L.S.G., M.O.L., J.M.M. Investigation: L.S.G., M.O.L., J.M.M. Writing – Original draft: L.S.G., M.O.L., J.M.M. Visualization: L.S.G., M.O.L., J.M.M. Supervision: L.S.G., M.O.L., J.M.M. Funding acquisition: LSG.

## Disclosure

This study was preregistered with the PROSPERO international prospective register of systematic reviews (Registration Number: CRD42024594627).

## Ethics Statement

The authors have nothing to report.

## Conflicts of Interest

The authors declare no conflicts of interest.

## Supporting information


**Data S1:** PRISMA—Reporting guideline checklist.

**Appendix A:** Search string in Pubmed.

**Appendix B:** Critical appraisal results for included studies.

## Data Availability

The data that support the findings of this study are available from the corresponding author upon reasonable request.
