# Supplementary material for: Interventions for Caregivers Caring for a Family Member With Advanced Illness at Home: A Systematic Review
Source: Nurs Health Sci. 2025 Aug 17;27(3):e70196. doi: 10.1111/nhs.70196 (PMC12358210; doi:10.1111/nhs.70196)
Supplement: Supplementary file 2 — Appendix A: Search string in Pubmed. [file NHS-27-e70196-s001.docx]

**Appendix A. Search string in Pubmed**

(((("Randomized Controlled Trials as Topic"[Mesh]) OR (((((((((Intervention[Title/Abstract]) OR (Strategies[Title/Abstract])) OR (Experimental[Title/Abstract])) OR (Program*[Title/Abstract])) OR ("Randomized Controlled Trial"[Title/Abstract])) OR ("Randomized Control Trial"[Title/Abstract])) OR ("Clinical trial"[Title/Abstract])) OR (Quasi-experimental[Title/Abstract])) OR ("Pre-post design"[Title/Abstract]))) AND (("Family"[Mesh]) OR (((((((((((Family[Title/Abstract]) OR (Families[Title/Abstract])) OR ("Family caregiver"[Title/Abstract])) OR ("Family caregivers"[Title/Abstract])) OR (Caregiver[Title/Abstract])) OR (Caregivers[Title/Abstract])) OR ("Family members"[Title/Abstract])) OR (Carer[Title/Abstract])) OR ("Informal caregivers"[Title/Abstract])) OR (Relatives[Title/Abstract])) OR ("Next of kin"[Title/Abstract])))) AND ((("Terminal Care"[Mesh]) OR ("Palliative Care"[Mesh])) OR (((((((("Terminal care"[Title/Abstract]) OR ("Palliative care"[Title/Abstract])) OR ("Hospice care"[Title/Abstract])) OR ("Advanced illness"[Title/Abstract])) OR ("End-of-life care"[Title/Abstract])) OR (EOL[Title/Abstract])) OR (Dying[Title/Abstract])) OR ("Last days"[Title/Abstract])))) AND ((("Home Environment"[Mesh]) OR ("Home Care Services"[Mesh])) OR (((((((("Home environment"[Title/Abstract]) OR ("Home care services"[Title/Abstract])) OR (Home[Title/Abstract])) OR (House[Title/Abstract])) OR (Housing[Title/Abstract])) OR (Dwelling[Title/Abstract])) OR (Residence[Title/Abstract])) OR (Home-based[Title/Abstract]))) Filters: English, Italian, Portuguese, Spanish, from 2019/1/1 - 2024/8/31

|  |
| --- |
