# Supplementary material for: Interventions for Caregivers Caring for a Family Member With Advanced Illness at Home: A Systematic Review
Source: Nurs Health Sci. 2025 Aug 17;27(3):e70196. doi: 10.1111/nhs.70196 (PMC12358210; doi:10.1111/nhs.70196)
Supplement: Supplementary file 3 — Appendix B: Critical appraisal results for included studies. [file NHS-27-e70196-s002.docx]

**Appendix B. Critical appraisal results for included studies**

**Table B1**

*Randomised clinical trials assessment using the Cochrane Risk of Bias 2 (RoB 2) tool (Sternet et al., 2019)*

| Citation | Q1 | Q2 | Q3 | Q4 | Q5 | Overall bias |
| --- | --- | --- | --- | --- | --- | --- |
| Dionne‐Odom et al. (2022) | L | SC | SC | SC | L | SC |
| Gregory & Gellis (2020) | SC | SC | L | L | L | SC |
| Lund et al. (2020) | L | L | SC | SC | L | SC |
| Mooney et al. (2023) | L | SC | L | SC | L | SC |
| Piamjariyakul et al. (2024) | L | L | SC | SC | L | SC |
| Valero-Cantero et al. (2023a) and Valero-Cantero et al. (2023b) | L | L | L | SC | L | L |
| von Heymann‐Horan et al. (2019) and von Heymann-Horan et al. (2023) | SC | SC | SC | SC | L | SC |

*Note.* L: low risk; SC: some concerns; H: high risk

**Table B2**

*Nonrandomised studies assessment using the Risk of Bias In Nonrandomized Studies of Interventions (ROBINS-I) tool (Sterne et al., 2016)*

| Citation | Q1 | Q2 | Q3 | Q4 | Q5 | Q6 | Q7 | Overall bias |
| --- | --- | --- | --- | --- | --- | --- | --- | --- |
| Boyko et al. (2021) | M | M | L | L | M | M | L | M |
| Chow et al. (2024) | M | M | L | L | M | M | L | M |
| Fleisher et al. (2023) | S | S | L | M | M | M | L | S |
| Ito & Tadaka (2022) | M | M | M | L | M | M | M | M |
| Norinder et al. (2024) | M | M | L | M | S | L | M | M |
| Petursdottir & Svavarsdottir (2019) | M | L | L | L | M | M | L | M |
| Rochmawati & Saun (2022) | M | M | L | M | L | M | L | M |

*Note.* L: low risk of bias; M: moderate risk; S: serious risk of bias; C: critical risk of bias; NI: no information
